# Medical Student Mobilization During a Pandemic: The Ochsner Clinical School Response to COVID-19

**DOI:** 10.31486/toj.20.0069

**Published:** 2020

**Authors:** Peter Ayoub, Donald D. Chang, Nadia Hussein, Kali Stewart, Amelia Wise, Iman Malik, Katherine Robbins, Bryan Savage, Melissa Johnson, Sangeeta Shah

**Affiliations:** ^1^ The University of Queensland Faculty of Medicine, Ochsner Clinical School, New Orleans, LA; ^2^ Division of Academics, Ochsner Clinic Foundation, New Orleans, LA; ^3^ Department of Cardiology, Ochsner Clinic Foundation, New Orleans, LA

## INTRODUCTION

In March 2020, the World Health Organization (WHO) declared that a novel coronavirus (COVID-19) had reached pandemic status. The United States experienced a rapid increase in cases that prompted statewide shutdowns, and hospitals quickly became overwhelmed with critically ill patients. Patient contact precautions evolved on a daily basis, and hospitals quickly depleted their stores of personal protective equipment (PPE). The unknown, but presumed highly infective, virus prompted hospitals to enact and enforce policies that forbade visitors from staying with their loved ones. Elective procedures and clinic appointments were paused until more information about the virus risk became available, keeping chronically ill patients from attending health maintenance appointments.

On March 17, 2020, the Association of American Medical Colleges (AAMC) issued a statement strongly encouraging the temporary suspension of medical school clinical rotations nationwide.^1^ The question of whether to withdraw students from patient care during a pandemic is a challenging one. An argument can be made that medical students should be well trained in the use of PPE and not shielded from the realities of patient care, including potential exposure to contagions.^2^ The decision becomes whether the educational benefits outweigh the risks of potential disease transmission from student contact with patients in the setting of limited PPE.

In the interest of student and patient safety, the Ochsner Clinical School (OCS), like medical schools across the United States, instituted a 2-week shutdown of clinical rotations to comply with the AAMC recommendation. With the students temporarily suspended from clinical duties, a valuable segment of the functional health workforce was suddenly underutilized. The American Medical Association published a public health newsletter detailing how medical students could assist during the COVID-19 pandemic; suggested activities included phone triage lines staffing, indirect patient outreach, and research.^3^ Similar activities, among others, were independently conceived by OCS students and faculty, and service projects were launched shortly after the clinical rotation pause began.

## IDENTIFICATION OF NEEDS

The students and faculty of the OCS focused their efforts on the needs of our Louisiana health system and identified the following: support triage efforts for community members suspected to have or concerned about COVID-19 infection, address the PPE shortage, assist with diagnostic efforts, discuss care with families separated from hospitalized patients, and ensure access to health maintenance appointments for disadvantaged patients.

### Triage Efforts

The COVID-19 pandemic raised significant concerns in the community about clinical and subclinical infection. The surge of inquiries placed significant stress on the established Ochsner triage call center. The sharp rise in call volume was reminiscent of the 2009 H1N1 pandemic when a Minnesota state call line fielded more than 27,000 calls.^4^ However, that Minnesota nurse triage line during the H1N1 pandemic was estimated to help prevent up to 11,000 hospital and clinic visits.^5^ The OCS administration determined that medical students could benefit the health system by fielding calls from community members, while maintaining minimal contact with patients and preventing unnecessary emergency department visits for those at risk of contracting the virus.

### Personal Protective Equipment Shortage

Because of the significant increase in the use of PPE by healthcare workers and community members alike, shortages in hospital PPE occurred. Early in the pandemic, the WHO estimated that PPE requirements would be 89 million medical masks, 76 million gloves, 30 million gowns, and 1.6 million pieces of eye protection each month.^6^ The combination of increased demand with disrupted supply chains created an urgent need to alleviate the strain on acquisition of necessary medical equipment.^7^ Medical students were ideal candidates to address PPE production and distribution concerns because of their knowledge of contact precautions and networking contacts within the health system.

### Diagnostic Testing

Some students had the credentials to serve in the specialized role of laboratory scientist to help conduct diagnostic testing on the large influx of SARS-CoV-2 samples. By order of the State of Louisiana Executive Department, potential laboratory personnel had to demonstrate real-time quantitative polymerase chain reaction (RT-qPCR) experience and/or demonstrate serologic experience in testing clinical samples.^8^ Two students who had the qualifications to perform the testing under the supervision of a lead pathologist volunteered in the laboratory.

### Community Outreach

The community outreach needs had two areas of focus. One centered around the need to inform patients’ families of treatment plans and patient clinical status because of the hospital limitations on visitors. The second area was to provide disadvantaged patients with the resources necessary to maintain their health and well-being. The Ochsner MedVantage Clinic was established in 2016 to assist moribund geriatric patients with multiple barriers to accessible medical care. Students and faculty determined that connecting with this established network of vulnerable patients could alleviate a predictable stress on the medical system. The medical students would assess and screen patients for signs and symptoms of deteriorating health, as well as facilitate the acquisition of basic needs such as food and safe housing.

## PROJECT DEVELOPMENT

A matrix-style organizational hierarchy was developed with levels of involvement and communication that would ensure project advancement (Figure). Lead faculty were in direct communication with project-specific faculty members and student project leaders who, in turn, communicated with each other. This hierarchic arrangement created a feedback loop that allowed for rapid adjustments and allocation of resources. Project leaders were tasked with refining the scope of each project, recruiting and scheduling student volunteers, and documenting service hours to be reported to OCS faculty.

**Figure.**
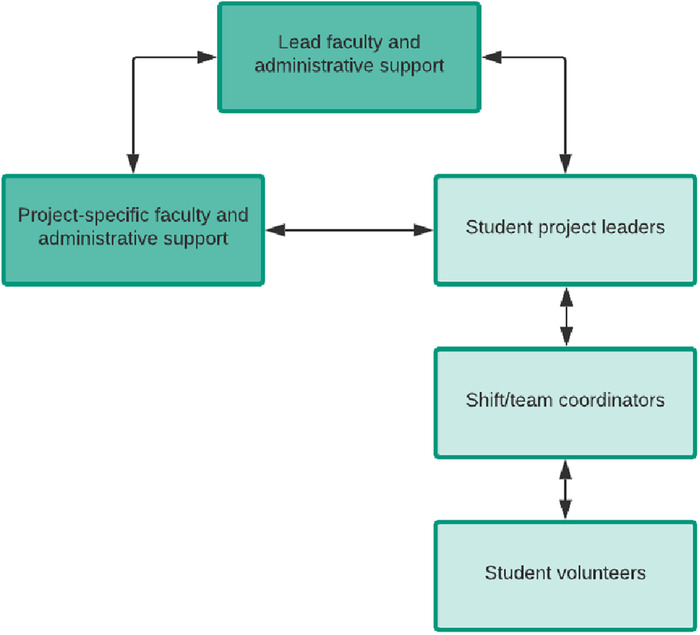
**Organizational hierarchy of Ochsner Clinical School faculty, student project leaders, and student volunteers.**

The OCS developed 6 projects: (1) COVID-19 Call Center, (2) Obstetrics (OB) COVID-19 Call Center, (3) Ochsner Medical 3-Dimensional (m3D) Lab PPE Initiative, (4) COVID-19 Diagnostic RT-qPCR Laboratory Testing, (5) MedVantage Clinic Telemedicine, and (6) Family Communication Champion Project (Table).

**Table.** Ochsner Clinical School Service Projects Developed During the COVID-19 Pandemic

Project	Description	Aims	Challenges
COVID-19 Hotline	Medical student–staffed triage hotline	Answer calls about symptoms concerns Inform citizens of shelter-in-place recommendations	Large volume of calls leading to lengthy wait times for patients
OB COVID-19 Call Center	Medical student–staffed triage hotline for current Ochsner obstetrics patients	Answer calls regarding concerns for the implications of COVID-19 on pregnancy Make outgoing calls to proactively check in on all current obstetric patients	Specialty patient population requiring tailored and up-to-date advice
Ochsner m3D Lab PPE Initiative	Intake, disinfect, assemble, and distribute face shields, hand-sewn masks, and gowns	Provide frontline workers with adequate PPE	Balance needs for significant manpower and social distancing
MedVantage Clinic Telemedicine	Clinic that assists geriatric patients with multiple barriers to healthcare access	Screen patients for depression, access to food and transportation, safe housing, sufficient medication Address most pertinent health issues, screen for COVID-19 symptoms, and advise patients on the importance of social isolation	Difficulties with helping elderly patients navigate electronic applications
Family Communication Champion Project	Student-led family communication service under physician direction	Relieve physician burden by updating families on patient clinical status and plan of care	Physician hesitation to entrust medical students with relaying sensitive information
COVID-19 Diagnostic RT-qPCR Laboratory Testing	Quantitative reverse transcription polymerase chain reaction (RT-qPCR) laboratory testing	Perform statewide SARS-CoV-2 diagnostic testing and report results	Immense workload because of relatively few qualified students

m3D, medical 3-dimensional; OB, obstetrics; PPE, personal protective equipment; SARS-CoV-2, severe acute respiratory syndrome coronavirus 2.

## IMPACT

A total of 151 student volunteers participated in the 6 COVID-19–related service projects, 71% of the student body of third- and fourth-year medical students. The service projects began on March 17, 2020 and continued through the 2-week suspension of clinical rotations, as well as through a 2-week period of limited clinical exposure that ended on April 12, 2020. The total number of volunteer hours completed in this 4-week time period was 3,625.

### Impact on the Community and Healthcare

Because of student staffing of the COVID-19 Call Center, caller wait times decreased from more than 3 hours with hundreds of callers in the queue to zero wait time with no callers on hold. The OB COVID-19 Call Center volunteers not only fielded incoming calls from pregnant and postpartum women across Ochsner Health but also spent significant time on patient outreach. The student volunteers proactively contacted 632 current OB patients to answer questions about COVID-19 during pregnancy, check for any developing symptoms, update patients on changing hospital policies, and distribute the direct phone number for the OB COVID-19 Call Center. Community members expressed their gratitude for these efforts, including this thank you from a 38-week pregnant, first-time mother: “…a quick, simple, easy phone call, but one that actually went a long way…that was comforting and reassuring during a time of such uncertainty.”

The MedVantage Clinic volunteers reached a total of 379 chronically ill geriatric patients and assisted them with accessing telemedicine communication options for health maintenance appointments.

Families and physicians provided positive feedback and appreciation for students who volunteered for the Family Communication Champion Project. Each student was assigned a physician and given a list of patient families to contact through either a secure chat instant messaging system or telephone. Information conveyed by students included the reason for admission, daily progress reports, and plan of care. Conversations with family members also provided a useful opportunity to gather collateral history for patients who were unable to provide information themselves. The time spent by students contacting families significantly reduced the workload of hospital physicians overwhelmed by the number of patients with COVID-19.

Ochsner led the state of Louisiana in COVID-19 samples testing, with more than 15,000 tests analyzed in 2 months. This quantity of RT-qPCR testing was, in part, made possible by OCS students with PhD-level training in laboratory settings.

Students who volunteered for the Ochsner m3D Lab PPE Initiative were responsible for the intake, disinfection, and distribution of tens of thousands of units of PPE to Ochsner medical facilities throughout the state of Louisiana. The Ochsner m3D Lab designed a novel face shield that was 3D printed by third-party manufacturers capable of high-volume production and delivered to the Ochsner m3D Lab PPE Initiative principal staging site. Hand-sewn gowns and masks from local textile manufacturers were also delivered to the staging site. All units of inventory were systematically disinfected using ultraviolet light, face shields were assembled, and all PPE was stored in disinfected bins to be delivered to Ochsner facilities. The face shields, gowns, and masks provided to frontline workers had a profound effect in preventing virus transmission among hospital workers and patients.

### Impact on Students

Other than academic achievement, key attributes characterize strong medical students who are equipped to excel in residency: trustworthiness, efficiency, being detail-oriented, professionalism, and being a self-directed learner.^9,10^ The service projects developed by OCS students and faculty allowed these characteristics to be demonstrated in an unprecedented manner: stepping up to answer a call to action during a national crisis. The opportunity to serve their community and bolster the healthcare workforce in nontraditional ways brought a great deal of pride to OCS students.

The knowledge students obtained during participation in the service projects took several forms. They learned (1) relevant medical knowledge by reviewing patient charts in detail, (2) the importance of nonphysician roles in healthcare teams, and (3) empathy and listening skills which were paramount in these uncertain times. The OCS service projects functioned as a surrogate for traditional clinical rotations by allowing students to use their established interpersonal clinical skills and medical knowledge to help close the gaps in healthcare when the system became overwhelmed.

Students also acquired and developed valuable leadership skills. The service projects provided situational experience with peer communication and task delegation. Students took the initiative to create structure and organization in their respective projects, using this opportunity to hone leadership skills that can be translated to future clinical practice.

## DISCUSSION

The lessons learned from disaster-based challenges produce protocols that allow for better preparedness in the event of future, similar challenges.^11^ Disaster events require and depend on the efforts of community leaders, first responders, hospital staff, and medical support workers.^12^ The concept of resilience centers around the adaptability of an individual or group of individuals to unexpected challenges.^13^ Medical students across the country showed their willingness to participate in service projects during a time of substantial need.^14^ The authors of this paper are in firm agreement that medical students can provide essential assistance to the medical workforce in the event of a major disaster or global crisis.

OCS students, with the support of faculty members, identified significant needs in the regional health system and filled those needs through coordinated efforts to establish meaningful service projects. We suggest that all medical programs be cognizant of the potential gaps in local healthcare and evaluate potential ways of bridging those gaps within a well-defined framework that prioritizes the needs of students. We recommend that faculty members work with student leaders to cosign on service projects and evaluate the projects for the potential of substituting course credit for hours served. This substitution of course credit would allow medical studies to remain effectively uninterrupted if clinical rotations are temporarily paused.

Of the 6 student service projects, 5 translated into clinical rotations once the OCS resumed routine clinical education. The COVID-19 Call Center was integrated into the Mental Health, Primary Care, and Medicine in Society (MIS) rotations. The OB COVID-19 Call Center was transitioned into the core OBGYN rotation for third-year students and used as elective credit for the OBGYN-interested fourth-year students. The MedVantage Clinic outreach efforts, a component of the MIS placement before the pandemic, was transitioned back to the MIS rotation but with a larger student capacity to permit more outreach and follow-up with high-risk patients. The Family Communication Champion Project was integrated into the third-year Internal Medicine rotation. The RT-qPCR Diagnostic Laboratory Testing project was transitioned into an elective placement for qualified students.

The interruption in student rotations, the strict patient contact precautions, and limited outpatient visits resulting from the COVID-19 pandemic strained traditional educational opportunities. Despite the reduced amount of conventional clinical experience, OCS students developed projects with relevance to a number of medical specialties, including internal medicine, obstetrics and gynecology, critical care, palliative medicine, infectious disease, geriatric medicine, and family medicine. Through strict documentation of student hours served on these projects, OCS faculty was able to assign course credit for students on rotations. The modifications have made it possible for students to return to clinical rotations and allowed for an uninterrupted school year.

## CONCLUSION

Third- and fourth-year medical students are at a junction where they are learning to transition from students to team leaders. Although institutions have an obligation to protect students from harm, a crisis such as the COVID-19 pandemic presents a unique opportunity to demonstrate critical thinking and clinical practice skills. Medical students are ideal candidates for identifying and addressing gaps in healthcare because of the training in fundamental patient care they have received throughout their time in medical school. Medical students nationwide have shown their determination and resilience through service projects during the COVID-19 pandemic. We expect to see similar tenacity in the event of future disasters.
